# Does the severity or cause of preoperative stiffness affect the clinical results and range of motion after total knee arthroplasty?

**DOI:** 10.1371/journal.pone.0205168

**Published:** 2018-10-11

**Authors:** Seung Ah Lee, Seung-Baik Kang, Chong Bum Chang, Moon Jong Chang, Young Jun Kim, Min Kyu Song, Jin Hwa Jeong

**Affiliations:** 1 Department of Physical Medicine and Rehabilitation, College of Medicine, Kyung Hee University, Seoul, South Korea; 2 Department of Orthopaedic Surgery, Seoul National University College of Medicine, SMG-SNU Boramae Medical Center, Seoul, South Korea; Consorci Parc de Salut MAR de Barcelona, SPAIN

## Abstract

The purpose of this study was to assess the overall clinical results and range of motion (ROM) after total knee arthroplasty (TKA) in patients with preoperative stiffness. We also aimed to determine whether the severity or cause of the stiffness can affect the clinical outcome after surgery. This retrospective study included 122 knees (117 patients) with follow-up of more than 2 years (mean age, 64.3 years). TKA was performed using posterior-stabilized, varus-valgus constrained (VVC), and hinged prostheses. To determine the effect of the severity of stiffness on the clinical outcome, the subjects were divided into two groups: the severe group (preoperative ROM ≤ 50°; 18 knees) and the moderate group (preoperative ROM, 50°–90°; 104 knees). Then, clinical results and ROM were compared according to the severity or cause of preoperative stiffness. After surgery, preoperative ROM (mean, 78°; range, 25°- 90°) was improved (mean, 107°; range, 70°- 130°). The severe group more frequently used the VVC or hinged prostheses (72% vs. 18%). Furthermore, the severe group had worse knee and function scores as well as more complications (33% vs. 13%), even though the severe group had a greater ROM increment (47° vs. 27°) after surgery. Patients with osteoarthritis and rheumatoid arthritis showed better ROM and clinical results compared to patients with infectious or traumatic arthritis. Although TKA in stiff knees can be successful, the results are inferior in knees with severe stiffness and knees with infectious or traumatic arthritis.

## Introduction

The main goals of total knee arthroplasty (TKA) are pain relief and functional improvement [1]. The majority of patients achieve these goals after surgery. However, a subset of patients are not satisfied with their surgical results [1]. In these patients, limited range of motion (ROM) after surgery can be one of the reasons for dissatisfaction [2–4]. Currently, in most studies, ROM is greater than in earlier reports, probably because of improved surgical techniques and newer prostheses [2,5]. However, postoperative ROM limitation still remains as an issue to be solved in TKA.

Postoperative ROM in patients with poor preoperative ROM may differ according to the severity of preoperative stiffness [6,7]. Generally, preoperative ROM is related to postoperative ROM [8–10]. Therefore, it is reasonable to speculate that postoperative ROM would be worse in patients with more severe preoperative stiffness. However, in other aspects, the severe stiff knees may have more chance to improve in terms of sheer degree of ROM. Thus, it is also possible to assume that the amount of ROM gain could be greater in patients with more severe preoperative stiffness. Therefore, an analysis of effects of severity of preoperative stiffness on the degree of postoperative ROM or ROM gain after TKA is required on a patient who had a preoperative stiffness.

Also, the causative disease of end-stage knee arthritis can affect clinical outcome after TKA. The stiffness caused by mechanical impingement can be resolved during surgery. However, adhesion involving soft tissues caused by a previous infection or trauma may be more difficult to fix [11]. Extensive soft tissue release can cause gap imbalance [12]. Thus, prostheses with more constraint should be used. These may result in a worse clinical outcome and less ROM gain after TKA. However, most previous studies focused only on reporting clinical outcome or complications after surgery [11,13,14], whereas others only compared differences in clinical outcomes between stiff knees and flexible knees [7]. Thus, multiple factors related to clinical outcome and ROM gain after TKA, including causes of preoperative stiffness, should be analyzed.

The purpose of this study was to assess the overall clinical results and ROM after total knee arthroplasty (TKA) in patients with preoperative stiffness. We also aimed to determine whether the severity or cause of the stiffness can affect the clinical outcome after surgery.

## Materials and methods

This retrospective study included 122 knees with follow-up of more than 2 years. From March 2005 to March 2012, 1859 TKAs were performed at our institution. We defined preoperative stiffness as a ROM of knee joint ≤ 90° [15]. Among the patients who underwent TKA, there were 134 knees (117 patients) with the ROM ≤ 90°. These 134 knees accounted for 7% of the total TKAs performed at our institution during that period. Of these, 122 knees (107 patients) with follow-up of more than 2 years were included in the present study. Twelve knees (9%) were excluded because they were lost to follow-up. The causes of stiffness were osteoarthritis in 80 knees (66%), rheumatoid arthritis in 12 knees (10%), infectious arthritis in 25 (20%), and traumatic arthritis in 5 (4%). No patient with previous infection had ongoing infection before TKA. The presence or absence of residual infection was screened using preoperative symptoms and laboratory tests including levels of erythrocyte sedimentation rate and C-reactive protein. Mean preoperative ROM was 78° (range, 25°- 90°). There were 25 males (29 knees) and 82 females (93 knees) with a mean age of 64.3 years (range, 43 years—83 years) at the time of surgery. Mean body mass index (BMI) of the patients was 27.1 kg/m^2^. This study protocol was approved by the Institutional Review Board of the SMG-SNU Boramae Medical Center (16-2016-132). All patients gave their consent for using and assessing their data.

All surgeries were performed using the same surgical protocol by a single senior surgeon (one of the authors), but various prostheses were used because of conditions of the knee joints and the surgeon’s preference. Spinal anesthesia was routinely tried for all patients, but in a subset of patients, general anesthesia was performed when the spinal anesthesia alone was not effective. A medial parapatellar approach with a tourniquet was used in most patients. However, a rectus snip was used to create a proper exposure in 27 knees (22%). The V-Y quadricepsplasty was used in only two (2%) knees, when a sufficient approach was not acquired using the rectus snip. No knee had a tibial tuberosity osteotomy. We used an intramedullary guide for resection of both the femur and tibia in all cases. If the knee was balanced and stable, a posterior-stabilized type implant was used. In contrast, if there was unacceptable soft tissue imbalance, a constrained prosthesis or a hinged prosthesis was used. All patellae were resurfaced and all components of the prosthesis were fixed with cement.

The rehabilitation protocol was the same except for patients with a V-Y quadricepsplasty. Continuous passive motion was started 2 days after surgery and the patients were encouraged to perform quadriceps strengthening exercises. Compression stockings and a pneumatic compression device were applied for thrombo-prophylaxis. Ambulation with a walker was allowed on the second postoperative day, as tolerated. Patients received inpatient physical therapy once daily for 20 minutes. They were instructed in home-based rehabilitation by therapist including ice, controlling swelling, walking and ROM. After discharge, they continue home exercise. In patients who underwent V-Y quadricepsplasty, the ROM was restricted with a brace in extension for 4 weeks. The active extension exercise and straight leg raises were withheld for 4 weeks. We did not perform manipulation under anesthesia postoperatively.

Data on clinical outcome were collected prospectively by a clinical investigator. Data on the causative disease of the end-stage arthritis and the prosthesis used were reviewed using the medial records. Clinical outcome including ROM, Knee Society (KS) knee score, KS function score, and Western Ontario and MacMaster (WOMAC) score were evaluated preoperatively and postoperatively at 6 months, 1 year and 2 years. The ROM of the knee joint was measured using a goniometer with the patient supine. Complications including wound necrosis, superficial infection, and deep infection were recorded. Recurrence of stiffness was defined as postoperative ROM ≤ 90° regardless of preoperative ROM.

Statistical analyses were performed using SPSS for Windows (version 18.0; SPSS Inc., Chicago, IL), and P values of <0.05 were considered statistically significant. Pre- and postoperative ROM and knee scores were described using mean and range. The statistical significance of the difference between pre- and postoperative data was determined using a paired t-test. Most previous studies defined preoperative stiffness as when the ROM was less than 50°, while other studies used 90° to define preoperative stiffness [6,7,15]. Thus, we divided the subjects into two groups: the severe group (preoperative ROM ≤ 50°; 18 knees) and the moderate group (preoperative ROM, 50°–90°; 104 knees). There was no difference in the causative disease of preoperative stiffness between the two groups (Table 1). In comparisons of the prostheses used between the two groups, number and percentage were used to describe the data, and the statistical significance of differences was determined using the chi-square test. For comparisons between the two groups with regard to the ROM and clinical scores, data were described using mean and range. The statistical significance of the difference between the two groups was determined using the Student t-test. To summarize the data for clinical results and ROM among the patients, according to the cause of preoperative stiffness, mean and range were used for knee scores and ROM. To describe the proportion of the knees that achieved a postoperative ROM ≥ 110°, number and percentage were used. The statistical significance was determined using analysis of variance (ANOVA) with post hoc analysis (Tukey method) and the chi-square test. A stepwise multiple linear regression analysis determined the effects of age, BMI, preoperative ROM and cause of stiffness on postoperative ROM. It was estimated that the sample size of the current study would be adequate to achieve a statistical power of 80% with < 5% probability of a type-I error.

**Table 1:** Comparisons of the cause of preoperative stiffness between the severe group and the moderate group.

Variable	Severe group	Moderate group	P value
Osteoarthritis	8 (44)	72 (69)	0.184
Rheumatoid arthritis	3 (17)	9 (9)	
Infectious arthritis	5 (28)	20 (19)	
Traumatic arthritis	2 (11)	3 (3)	

Data are presented as number and percent in the parentheses.

## Results

Clinical outcome after TKA in patients with stiff knees improved after surgery in terms of ROM and knee and function scores (Table 2). Preoperative ROM (mean, 78°; range, 25°- 90°) substantially improved after surgery (mean, 107°; range, 70°- 130°). The mean amount of ROM increment was 30° (range, 0°- 60°), and 58 knees (48%) had ROM greater than 110°. Stiffness recurred in seven knees (6%). The clinical scores evaluated including KS knee scores, functional scores and WOMAC total scores were substantially improved. The mean KS knee score was improved from 43 points before TKA to 77 points after surgery. The mean WOMAC total score was also improved from 43 points to 21 points after surgery.

**Table 2:** Overall clinical results.

Variable	Preoperative	Postoperative	P value
Range of motion (°)	78 (25–90)	107 (70–130)	< 0.001
KS knee score (points)	43 (15–73)	77 (52–94)	< 0.001
KS function score (points)	42 (5–70)	76 (51–93)	< 0.001
WOMAC total score (points)	43 (9–76)	21 (8–41)	< 0.001

Data are presented as mean and range in the parentheses. Abbreviations: KS = Knee Society, WOMAC = Western Ontario and MacMaster

Total knee arthroplasty in the severe group used prostheses with more constraint and had worse knee and function scores as well as more complications even though patients in the severe group had a greater ROM increase after surgery. During surgeries, TKA in the severe group used VVC or hinged prostheses in 72% whereas TKA in the moderate group used these prostheses only in 18% of knees (Table 3). In contrast to the greater ROM increment, the severe group showed a similar amount of improvement in knee and function scores. The knee and function scores after surgery in the severe group were inferior compared to those of the moderate group because the severe group had lower scores before surgery (Table 4). Recurrent postoperative stiffness developed more frequently in the severe group than the moderate group (33% vs. 1%). Furthermore, the severe group had more complications (Table 4; 33% vs. 13%). One of the six complications in the severe group was pyogenic arthritis, whereas the majority of complications in the moderate group were superficial wound infections (11 of 13 knees).

**Table 3:** Comparisons of the prosthesis type used between the severe group and the moderate group.

Variable	Severe group (n = 18)	Moderate group (n = 104)	P value
PS prosthesis	5 (28)	85 (82)	< 0.001
VVC prosthesis	11 (61)	18 (17)	
Hinged prosthesis	2 (11)	1 (1)	

Data are presented as number and percent in the parentheses. Abbreviations: PS = posterior stabilized, VVC = varus-valgus constrained

**Table 4:** Comparisons of clinical outcome between the severe group and the moderate group.

Variable	Severe group (n = 18)	Moderate group (n = 104)	P value
Range of motion (°)			
Preoperative	45 (25–50)	82 (55–90)	< 0.001
Postoperative	92 (70–110)	110 (85–130)	< 0.001
Amount of increment	47 (35–60)	27 (0–60)	< 0.001
KS knee score (points)			
Preoperative	36 (25–49)	44 (15–73)	< 0.001
Postoperative	67 (52–92)	79 (51–93)	< 0.001
Amount of increment	31 (15–59)	35 (0–76)	0.210
KS function score (points)			
Preoperative	35 (20–50)	43 (5–70)	< 0.001
Postoperative	69 (58–84)	78 (51–93)	< 0.001
Amount of increment	34 (23–44)	35 (5–79)	0.886
WOMAC total score (points)			
Preoperative	51 (45–59)	42 (9–76)	< 0.001
Postoperative	31 (18–41)	20 (8–41)	< 0.001
Amount of changes	20 (10–33)	21 (9–55)	0.692
Recurrence of stiffness (ROM ≤ 90°)	6 (33)	1 (1)	< 0.001
Complications	6 (33)	13 (13)	< 0.001
Superficial infection	3	11	
Deep infection	1	1	
Skin necrosis	2	1	

Data are presented as mean and range in the parentheses except for the recurrence of stiffness and the complications. Data of the recurrence of stiffness and the complications are presented as number and percent in the parentheses. Abbreviations: KS = Knee Society, WOMAC = Western Ontario and MacMaster, ROM = range of motion

Total knee arthroplasty in patients with preoperative stiffness caused by osteoarthritis and rheumatoid arthritis showed better ROM and clinical results compared to patients with infectious or traumatic arthritis. Mean ROM was greater than 110° in patients with osteoarthritis and rheumatoid arthritis after surgery, whereas only 13% (3 of 25 knees) with infectious arthritis achieved a ROM greater than 110° (Table 5). Clinical results were also better in patients with osteoarthritis and rheumatoid arthritis.

**Table 5:** Comparisons of pre- and postoperative range of motion and clinical outcome according to the cause of preoperative stiffness.

Variable	OA (n = 80)	RA (n = 12)	Infectious arthritis (n = 25)	Traumatic arthritis (n = 5)	P value
Preoperative	79 (25–90)	74 (35–100)	72 (35–90)	71 (45–90)	0.224
Postoperative	110 (80–130)	112 (95–130)	96 (70–130)	97 (80–110)	<0.001^§^
^ǂ^Patients with ROM ≥ 110°	46 (57)	8 (67)	3 (13)	1 (20)	<0.001
KS knee score (points)					
Preoperative	45 (15–73)	42 (28–55)	36 (29–52)	39 (30–48)	0.026*
Postoperative	80 (52–94)	82 (65–89)	66 (55–93)	68 (58–78)	<0.001^#^
KS function score (points)					
Preoperative	44 (5–70)	38 (28–60)	36 (15–50)	41 (32–50)	0.067
Postoperative	79 (61–91)	82 (65–89)	65 (51–81)	68 (58–78)	<0.001^†^
WOMAC total score (points)					
Preoperative	42 (9–76)	47 (38–57)	44 (22–70)	46 (33–56)	0.603
Postoperative	20 (8–40)	19 (13–28)	28 (15–41)	28 (17–38)	<0.001^‡^

Data are presented as mean with range in parentheses.

^ǂ^Data are expressed as number and percent in parentheses.

Post hoc analysis:

^§^Osteoarthritis vs. infectious arthritis, p < 0.001.

*Osteoarthritis vs. infectious, p = 0.020.

^#^ Osteoarthritis vs. infectious, p < 0.001; Osteoarthritis vs. traumatic, p = 0.004; Rheumatoid vs. infectious, p < 0.001; Rheumatoid vs. traumatic, p = 0.006.

^†^Osteoarthritis vs. infectious, p < 0.001; Osteoarthritis vs. traumatic, p = 0.003; Rheumatoid vs. infectious, p < 0.001; Rheumatoid vs. traumatic, p < 0.001.

^‡^Osteoarthritis vs. infectious, p < 0.001; Rheumatoid vs. infectious, p = 0.002. Abbreviations: OA = osteoarthritis, RA = rheumatoid arthritis, KS = Knee Society, WOMAC = Western Ontario and MacMaster

Multiple regression analysis (adjusted R^2^ = 0.362) showed that preoperative ROM (beta = 0.443; p <0.001) and cause of stiffness (beta = -0.363; p <0.001) were significant factors related to postoperative ROM. In simple terms, patients who had better preoperative ROM showed greater postoperative ROM. Also, patients with OA and RA showed greater postoperative ROM than patents with infectious and traumatic arthritis (Table 6).

**Table 6:** Comparison of the standardized beta values from the multiple regression analysis.

Variable	Standardized beta	p-value
BMI (kg/m^2^)	-0.145	0.051
Preoperative ROM	0.443	<0.001
Cause of stiffness	-0.363	<0.001

Abbreviations = BMI, body mass index; ROM, range of motion

## Discussion

In the present study, we hypothesized that clinical outcomes including ROM, knee score, and function score could be substantially improved in contemporary TKA despite stiffness (ROM ≤ 90°) before TKA. We also hypothesized that clinical outcome would differ according to the severity and causative disease of preoperative stiffness. The principal finding of this study was that TKA substantially improved ROM and functional scores in the patients with preoperative stiffness. We also found that the severe stiffness group had more ROM gain than the moderate group. Nonetheless, the final ROM and clinical scores of the severe group were inferior, with a higher complication rate than in the moderate group. In addition, the stiffness caused by infectious and traumatic arthritis was associated with worse clinical outcomes after surgery. In multiple regression analysis, preoperative ROM and cause of stiffness were related to postoperative ROM. Our findings suggest that TKA can improve ROM and functional scores even in the presence of severe preoperative stiffness, but surgeons should be cautious about inferior outcome in patients with more severe preoperative stiffness and the stiffness caused by infectious and traumatic arthritis.

Our findings are in line with the findings of previous studies showing that TKA improved ROM and functional scores in patients with poor preoperative ROM [16–18]. McAuley et al. reported that an average preoperative ROM of 30° (range, 0°– 50°) was improved postoperatively to an average ROM of 74° (range, 15°– 110°) after TKA [18]. Even in fused knees, substantial improvement of ROM (more than 60° flexion after TKA) has been reported [6,13,19–23]. However, it is uncertain what degree of ROM is required to satisfy the patients’ expectations. It is reported that ROM around 110° flexion is needed to perform daily activities [2]. Even though most patients with preoperative stiffness obtained substantial ROM gain after surgery, only a subset of patients was able to achieve this ROM. In the present study, 48% of knees had ROM greater than 110°. Previously, only 29% of patients with previously ankylosed knees were satisfied after surgery, even if they achieved mean flexion of 62° after TKA. Although TKA can improve ROM and functional outcome in patients with preoperative stiffness, it may not be enough compared to patients’ expectations. Therefore, clinicians should give information to their patients regarding the limitation in postoperative ROM gain and functional improvement. In addition, surgeons should counsel patients to have realistic expectations.

The results of this study support the hypothesis that clinical outcome and ROM differ according to the severity of preoperative stiffness. In the previous comparison between stiff and flexible knees, mean ROM gain was greater in the stiff knees: an increment of 64.4% in stiff knees vs. 59.9% in the flexible knees [15]. In the present study, we found that the severe group had more ROM gain than the moderate group. Nonetheless, the final ROM and clinical scores of the severe group were inferior, with a higher complication rate than in the moderate group. With regard to the ROM increment in stiff knees, it has been believed that a knee with limited ROM in flexion has a better prognosis than does one with limited ROM in extension [6,24]. A previous study, using 27 knees in 24 patients with spontaneous bony ankylosis in severe flexion (mean posture, 105° flexion), reported an average ROM of 91° postoperatively. The mean ROM of this previous study is greater than those in other studies that dealt with ankylosed knees [6,19,25]. We speculated that it is related to the relatively longer length of the extensor mechanism of knees ankylosed in flexion than knees ankylosed in extension. Joint replacement in knees that had stiffness in the extension position through TKA can result in increasing the tension of the soft tissues in the front of the knee. Therefore, proper surgical technique to lengthen the extensor mechanism of the knees is important to obtain maximal flexion [24]. Fortunately, we did V-Y quadricepsplasty in only two knees, probably because the subjects of this study had relatively greater ROM compared to those in other studies that included ankylosed knees [13,22,23,26,27].

Our finding also affirms the hypothesis that clinical outcome would differ according to the cause of preoperative stiffness. Aglietti et al. compared ROM between patients with ankylosed knees caused by osteoarthritis and those caused by rheumatoid arthritis. The mean arc of motion was the same at 78° in both groups after TKA [6]. In contrast to the reasonable outcome in rheumatoid arthritis, poor postoperative ROM was reported in patients with ankylosing spondylitis [27]. In the previous series, there was no increase in ROM after 2 years. The authors explained the causes of the inferior ROM using three reasons: 1) the nature of the disease that causes soft-tissue contracture and joint ankylosis, 2) poor preoperative ROM, and 3) the relatively high rate of heterotopic ossification. On the other hand, it was shown that patients with post-traumatic arthritis more frequently needed V-Y quadricepsplasty and then achieved poor postoperative outcomes [10]. With regard to infectious arthritis, there was a study that reported the reasonable restoration of function after TKA: mean postoperative ROM of 75.3° in knees with complete, and 98.7° in those with partial ankylosis [11]. However, in the present study, knees with infectious arthritis showed less ROM and lower functional scores. We speculate that the soft tissue scarring caused by longstanding infection and multiple operations probably affected postoperative ROM.

This study has several limitations. First, the study was retrospectively performed and there was no control group of flexible knees. However, we think this study can provide valuable information to readers by comparing knees with severe stiffness to knees with moderate stiffness, which are encountered more frequently during clinical practice. Second, the follow-up period of more than 2 years was relatively short. Thus, we were not able to examine complications such as aseptic loosening of the prosthesis or late prosthetic joint infection, which can develop in the longer term after surgery. However, we believe that the two-year follow-up was enough to present postoperative ROM and knee scores because the postoperative ROM rarely increases one year after surgery [15].

## Conclusions

Total knee arthroplasty improves ROM and clinical results after surgery in patients with preoperative stiffness. However, the clinical outcome was inferior in knees with more severe preoperative stiffness or stiffness caused by secondary arthritis such as infectious arthritis and traumatic arthritis. For those patients, special measures such as active physical therapy may be necessary. In addition, the findings of this study should be used to counsel patients before performing TKA in knees with preoperative stiffness.

## Supporting information

S1 Appendix Row data set used in the study. The entire dataset used in this study is presented in the file S1 Appendix.
(XLSX)
